# Laser Active Optical Systems (LAOSs) for Material Processing

**DOI:** 10.3390/mi16070792

**Published:** 2025-07-02

**Authors:** Vladimir Chvykov

**Affiliations:** Department of Electrical and Computer Engineering, Colorado State University, Fort Collins, CO 80523, USA; vchv@colostate.edu

**Keywords:** lasers, laser material processing, laser amplifiers, active optical systems

## Abstract

The output energy of Laser Active Optical Systems (LAOSs), in which image brightness is amplified within the laser-active medium, is always higher than the input energy. This contrasts with conventional optical systems (OSs). As a result, a LAOS enables the creation of laser beams with tailored energy distribution across the aperture, making them ideal for material processing applications. This concept was first successfully implemented using metal vapor lasers as the gain medium. In these systems, material processing was achieved by using a laser beam that either carried the required energy profile or the image of the object itself. Later, other laser media were utilized for LAOSs, including barium vapor, strontium vapor, excimer XeCl lasers, and solid-state media. Additionally, during the development of these systems, several modifications were introduced. For example, Space-Time Light Modulators (STLMs) and CCD cameras were incorporated, along with the use of multipass amplifiers, disk-shaped or thin-disk (TD) solid-state laser amplifiers, and other advancements. These techniques have significantly expanded the range of power, energy, pulse durations, and operating wavelengths. Currently, TD laser amplifiers and STLMs based on Digital Light Processor (DLP) technology or Digital Micromirror Devices (DMDs) enhance the potential to develop LAOS devices for Subtractive and Additive Technologies (ST, AT), applicable in both macromachining (cutting, welding, drilling) and micro-nano processing. This review presents comparable characteristics and requirements for these various LAOS applications.

## 1. Introduction

Laser systems used in manufacturing are generally classified into two main categories based on their operating regimes: Continuous Wave (CW) operation, where the laser is continuously pumped and emits a steady beam of light, and the pulsed regime, in which the laser generates single or multiple pulses at varying repetition rates [1].

Various laser active media are employed in these systems, including solid-state (crystals or glass) disk lasers [2], high-power fiber lasers created from active optical fibers pumped by semiconductor laser diodes, and diode lasers themselves. These laser media have enabled the achievement of impressive output powers of up to hundreds of kilowatts [3], a broad range of operating wavelengths, and wall-plug efficiencies of up to 50% [4]. Multi-kilowatt carbon dioxide (CO_2_) gas lasers are another example, offering greater reliability and higher output consistency, which has led to their widespread use in industrial applications [5].

**Figure 1:**
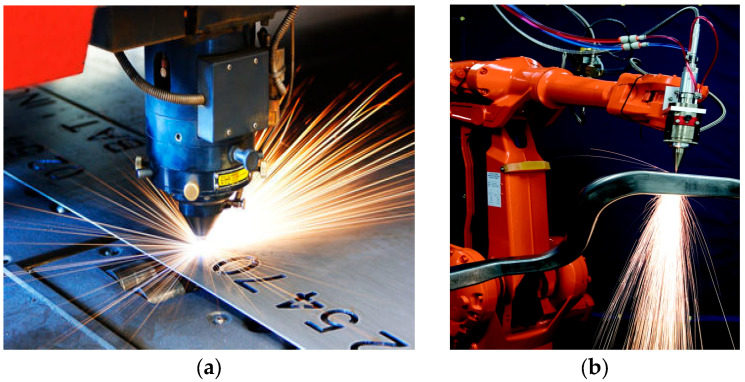
(**a**) The operation of 2D laser scanner (Copyright 2006 The Gale Group Inc.); (**b**) the operation of 3D laser manipulator (Bill Shiner, Delivering power, Nature Photonics, 2010, 4, 290).

Most conventional laser devices designed for material processing generate high-power light beams with Gaussian or flat-top beam profiles, which are then focused and scanned across the material surfaces. This laser-based material processing is utilized in both 2D and 3D Subtractive Technology (ST) (Figure 1), as well as in Additive Technology (AT).

**Figure 2:**
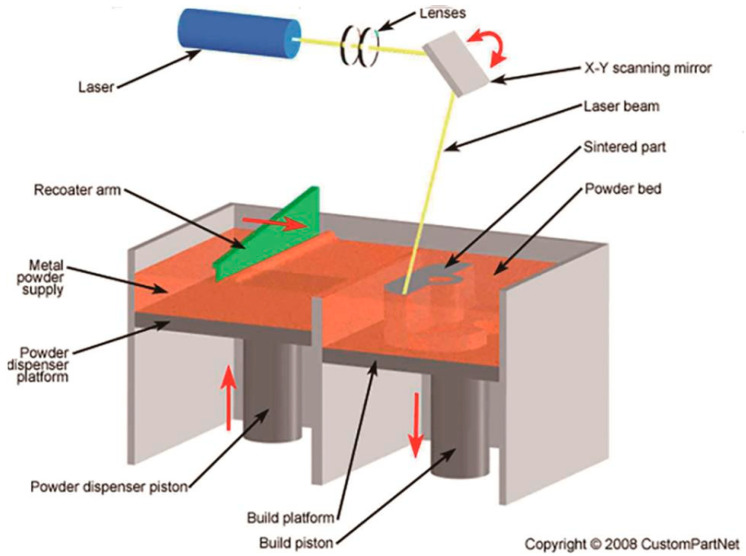
Scanned laser beam used in AT.

In ST, powerful laser systems are used for traditional industrial machining. Numerous publications discuss the technological aspects of processes such as drilling [6], cutting [7], welding [8], and surface modification [9]. On the other hand, in AT, a scanned laser beam is used to sinter material powder in specific areas, layer by layer, to build a three-dimensional object (see Figure 2) [10]. This technology is one of the main trends in modern manufacturing and is expected to replace 80–90% of traditional ST processes within the next 10–20 years, according to experts. AT is already being applied across a wide range of industries, from household goods to aircraft engines, and utilizes materials ranging from plastics to various types of high-strength steel reinforcement.

For both ST and AT, the manufacturing process requires scanning the focused laser beam during treatment, which necessitates complex mechanical scanning and beam delivery systems. These systems significantly reduce the speed and flexibility of manufacture. This issue is especially critical for current AT systems, limiting their potential for mass production.

A more effective technique involves using a defocused and structured light beam to simultaneously expose large areas of the material surface. This can be done in two different ways. One method is employed in AT, when low light power is sufficient for material sintering [11]. In this approach, for each slice of the object, the light beam reflected from the DMD projects a patterned light that selectively exposes and hardens the resin.

Since an entire layer is exposed by a single pattern, fast build speeds are achieved, regardless of the layer’s complexity. Another method for obtaining a structured beam involves introducing a patterned mask into the uniform beam. One of the key applications of this approach is in the microchip manufacturing industry [12]. However, in both of these methods, the light power is constrained by the damage threshold of the DMD or masks, as well as energy losses in the passive optical systems, where the output brightness of the image is always lower than the input.

**Figure 3:**
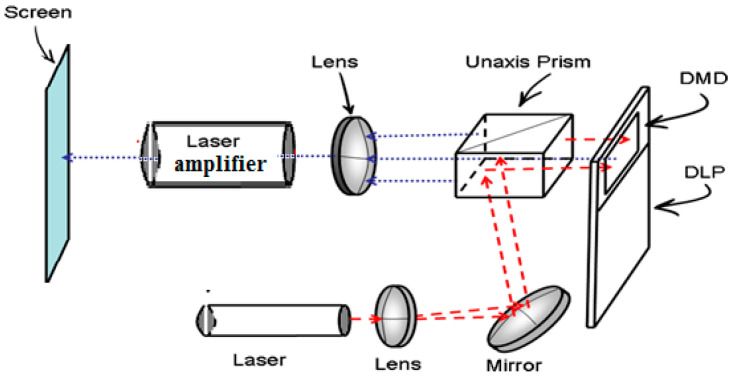
LAOS with the laser amplifiers incorporated into DLP projector (Modified from [13]).

This situation was improved with Laser Active Optical Systems, where the output energy of the beam carrying the corresponding energy profile is higher than the input, allowing the fluence on the Spatial Temporal Light Modulator (STLM) to remain below the damage threshold. The schematic of this main idea is presented in Figure 3, which illustrates LAOS laser amplifiers integrated, for example, into a DLP projector. In this scheme, laser light passes through an optical system consisting of a lens, mirror, and prism, illuminating the DMD (red dashed arrows). The reflected beam, carrying the image with opposite polarization, passes through the prism and projection lens being directed onto the screen through the laser amplifier, where its energy was increased (blue doted arrows). A more detailed explanation of LAOSs development from a historical retrospective will be presented.

## 2. LAOSs

**Figure 4:**
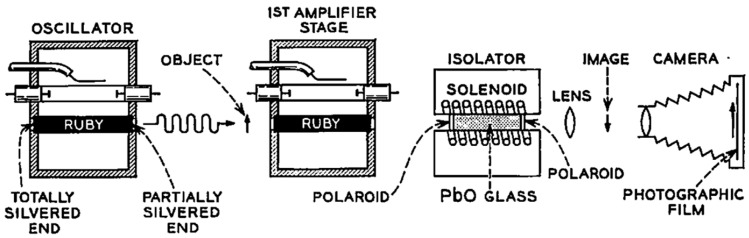
Schematic description of the image amplification experiment [14].

One of the earliest attempts to use a laser for image brightness amplification was made in 1962 by J. E. Geusic and H. E. D. Scovil [14] soon after lasers were invented using ruby laser amplifiers. The scheme of the oscillator- amplifier was used in this case, which allowed obtaining a gain of 5db. The object was illuminated by the oscillator and the transmitted light directed into the amplifier with an active element of 0.2”x3”. The author used the ruby rod as a limited aperture and an infinite focal length lens with gain. To show that an image could be sent through this rod and amplified, a projection slide having on it a number of dark lines was placed in the beam before the input of the amplifier. The slide was then viewed with a lens and a camera, as shown in Figure 4. The amplified image had very poor quality, which is why this system did not reach further development (J. E. Geusic and H. E. D. Scovil, Bell Syst. Tech. J. 41, 1371 (1962)).

Similar results and the image quality were obtained, supported by experiments using a helium–neon gas laser (HeNe laser) [15]. This study described an optical cavity where the modes are defined as stationary states of the diffraction-limited object/image transformation in classical optics. The study explored the properties of these modes in both small and large optical aperture limits.

**Figure 5:**
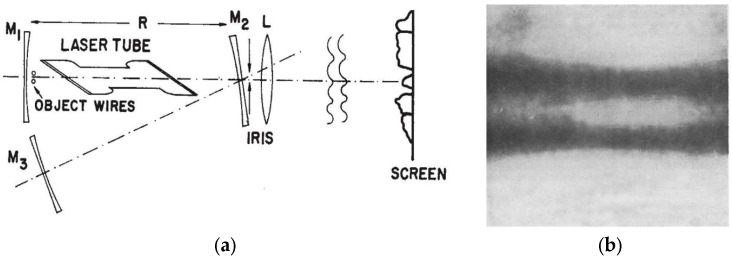
(**a**) Schematic diagram of the active image formation in the cavity of HeNe laser; (**b**) amplified image of two parallel wires in active formation [15].

The mode degeneracy of the optical cavity shown in Figure 5 is utilized in image formation, where opaque objects placed in front of mirror M1 influence the resulting field distributions in laser oscillation. As a result, the light generated within the cavity and partially transmitted through one of the mirrors can be used to reimage the object. In Figure 5a, mirrors M1, M2, and M3 all have an equal radius of curvature of 190 cm and are coated for high reflectivity—99% at 632.8 nm.

A gas discharge tube, excited over a 70 cm length with an inner diameter of 0.7 cm, was used in the setup. Within the optical cavity, near the surface of mirror M1, an object consisting of two parallel wires—each 25 μm in diameter and separated by 75 μm at their centers—was positioned. This object was reimaged onto the screen through partially transmitting mirror M2 by lens L. Mirror M2 functions as an objective lens, enabling repeated imaging of mirror M1’s surface onto M3, and then reimaging M3 back onto M1. This process facilitates the multiple reflections required for laser oscillation. The degraded quality of the reconstructed image, shown in Figure 5b, is attributed to the limited formation of resonant modes within the laser cavity.

**Figure 6:**
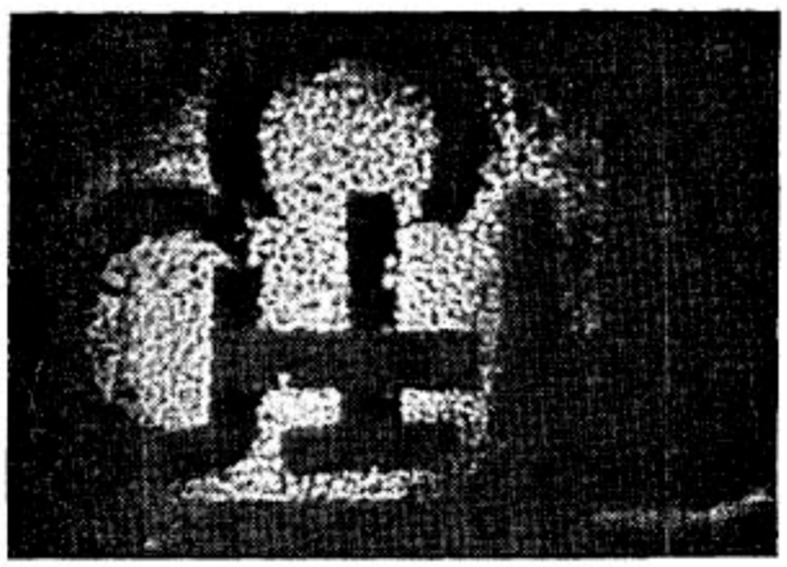
Image with amplifying through ruby lens and projection optics [16].

Another attempt to enhance image brightness and quality involved using a thick ruby lens as a coherent amplifier, as demonstrated by E. R. Lancz [16]. A ruby active medium, shaped as a convex lens with a radius of curvature of 2.74 cm, a thickness of 6.35 cm, and a diameter of 9.5 mm, was employed as the amplifying element. This amplifier lens was paired with a converging glass lens to create the projection optical system. An amplification factor of 2 was achieved by transmitting and amplifying the good quality images through this lens, as shown in Figure 6.

However, these early efforts resulted in low image quality or limited amplification, preventing further development and practical applications. A more significant advancement in LAOSs was achieved with the use of metal vapor laser (MVL) active media as image brightness amplifiers [17,18]. This development led to the creation of several LAOSs for micro- and macromachining.

The development and mass production of MVLs featuring sealed-off active elements, with lifetimes extending to thousands of hours, paved the way for the creation of this new LAOS type. These innovations made it possible to refine the parametric ranges of laser active elements, offering output average powers from 1 to 100 W [19,20,21,22,23,24,25,26,27,28]. The key parameters of the manufactured lasers and active elements are listed in Table 1 [27].

**Figure 7:**
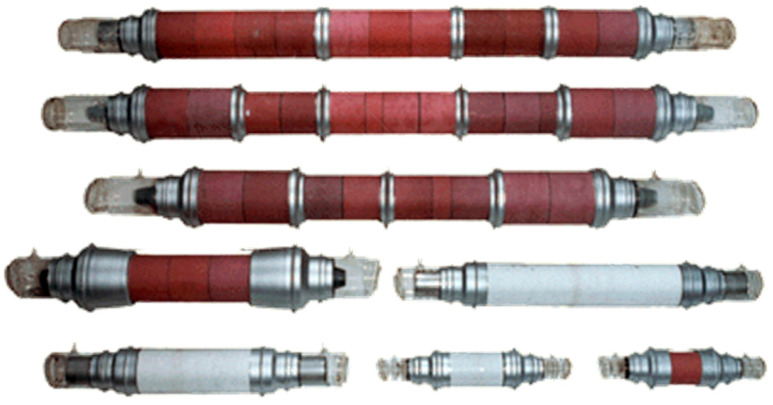
Commercially available MVL laser elements [27].

Some of these sealed-off active elements [27] are presented in Figure 7. The large internal diameter of copper vapor laser (CVL) tubes allows for average power levels reaching up to hundreds of Watts (see also Figure 7, two upper elements).

CuBr lasers with H_2_ and copper HyBrID lasers have demonstrated average powers exceeding 100 W, with an efficiency of 2–3%, which is higher than that of CVLs.

Additionally, specialized copper vapor laser tubes have been designed specifically for use as amplifiers in projection and micromachining systems [29]. When used as optical amplifiers, the active elements of these lasers can provide sufficient energy to illuminate a large area of the processing surface. Their properties—such as high gain, high repetition rates, operation across different spectral ranges, and high resolution—make them well suited for integration into LAOSs.

Table 2 presents the main parameters of metal vapor image brightness amplifiers measured in relatively small devices, with laser discharge tube inner diameters of 2 cm or less.

The average output power of nearly all lasers with active media listed in Table 2 exceeds 10 W.

The first operational LAOS scheme utilizing these types of amplifiers was a laser projection microscope. This device, along with other practical LAOSs, was developed and studied in the laboratory of G.G. Petrash at the Lebedev Physical Institute in Russia.

**Figure 8:**
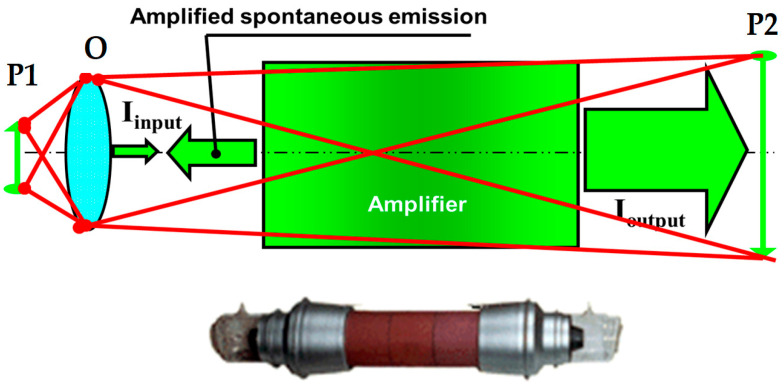
Optical system of laser projection microscope.

The key operational characteristics of the projection microscope were discussed in detail in [18]. It was demonstrated that a projection system intended to produce images with a linear magnification of 1000, while maintaining adequate brightness on a large screen, necessitates a light flux on the object that is at least 10^7^ times greater than the minimum required illuminance of the screen. The challenge of achieving adequate screen illuminance led to the need for a novel approach. The solution was found by incorporating amplifying elements, similar to the active components used in lasers. A schematic of the simplest laser projection microscope with an amplifying active medium is shown in Figure 8. In this design, the active medium serves both to illuminate the object and to amplify the image-carrying beam. The primary distinguishing characteristics of these active media were outlined as follows:

▪ Optical Homogeneity: The active medium must be optically uniform to prevent introducing significant distortions into the image.
▪ High Single-Pass Gain: The active medium should exhibit a sufficiently high real single-pass gain to substantially enhance image brightness.
▪ Moderate Gain, Sufficient Power: Rather than being of a high-gain type, the active medium should provide a sufficiently high average power to achieve adequate screen illuminance for viewing.
▪ Spectral Range for Direct Viewing: If the image is to be viewed directly, the active medium should operate within the visible spectrum, either continuously or in pulsed mode.
▪ Adequate Dimensions and Angular Aperture: The size and angular aperture of the active medium must be large enough to accommodate all image-carrying light rays.
▪ High Laser Efficiency and Saturation Operation: The active medium should possess high laser efficiency and operate close to saturation, ensuring optimal overall system performance.

Meeting all these requirements simultaneously proved challenging, and only a limited number of lasers available at the time were suitable for use in a Laser Projection Microscope (LPM) with parameters of practical significance. Among the various options, pulsed lasers utilizing active media such as copper, lead, barium, manganese, or gold vapor (see Table 2 and [30]) were found to best satisfy these conditions. Even in the early experiments, copper vapor amplifiers were able to produce bright, high-quality amplified images with a linear magnification of up to 15 × 10^3^. Subsequently, linear magnifications of up to 10^5^ were achieved.

The typical optical scheme of the LPM is shown in Figure 8. The object was positioned in plane P1 and illuminated by Amplified Spontaneous Emission (ASE) from the active medium through an objective (O). ASE refers to the amplification of spontaneously emitted photons within the laser medium. In the presented setup, ASE primarily consists of photons that are emitted and subsequently amplified from the section of the amplifier opposite to the object, due to the longer available path for amplification. Light reflected from the object was collected by the objective and, after amplification in the active medium, formed an image on a screen in plane P2, which was conjugated to plane P1. The red lines in the diagram represent beams originating from two distinct points on the object, which propagate through the amplifier and form the image. The magnification of this system was determined by the ratio of the distances OP_2_/OP_1_ and could be significantly increased if the brightness was sufficiently high. The image brightness depended on the parameters of the amplifying medium. Below, the image shows the commercial amplifier used for this system.

**Figure 9:**
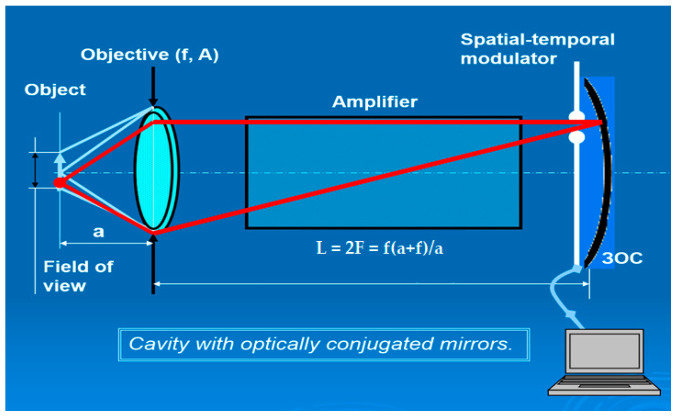
LAOS with conjugate resonator.

If a reflector is introduced into this optical system to direct the light beam back to the object through the amplifying element, a resonator can be formed (see Figure 9) [29]. In this resonator, the object itself can serve as a second reflector. The addition of a feedback reflector allows for controllable illumination of the object, enhancing the system’s performance.

A schematic diagram of a projection system with an amplifier and a conjugate resonator is shown in Figure 9. In this configuration, the object and the feedback mirror (the two reflectors of the resonator) are positioned in optically conjugated planes. The objective lens forms an image of the object on the feedback mirror and vice versa. The feedback mirror is spherical, with a radius of curvature equal to the distance between the mirror and the objective. In this resonator, any ray originating from a point on the object is returned to the same point after reflection from the feedback mirror. Amplified image projection in this system can be achieved either through a semi-transparent mirror or by using a beamsplitter with additional projection optics. This setup enables the formation of an intense beam that illuminates the entire field of view on the object, potentially leading to a significant increase in output power. Placing a mask in front of the feedback mirror allows selective illumination of the object, restricting light to the areas corresponding to the open sections of the mask, enabling precise processing. The STLM was used as a mask, as shown in the picture.

A conjugate resonator creates a complex intensity distribution on the object, replicating the pattern of the mask. By using the STLM, the light pattern on the object was electronically controlled, significantly enhancing processing efficiency [31,32,33]. As shown in Figure 9, the object to be processed is positioned as one of the resonator’s reflectors.

In a resonator where the image on the object is strongly demagnified, the power density becomes significantly higher than on the STLM, enabling processes such as ablation. Notably, the intensity of light beams within the resonator depends on the reflectivity of the object. This allows for precise processing, such as selectively removing a highly reflective layer while preserving an underlying transparent substrate.

If a suitably partially transparent mirror is used instead of the object, a desired light pattern can be formed on the object through an additional optical system that projects this mirror onto the object. This approach makes the processing independent of the object’s reflectivity. However, a drawback of these systems is the increased illumination of the mask, which may lead to its damage. As a result, these LAOS configurations are suitable only for micromachining.

**Figure 10:**
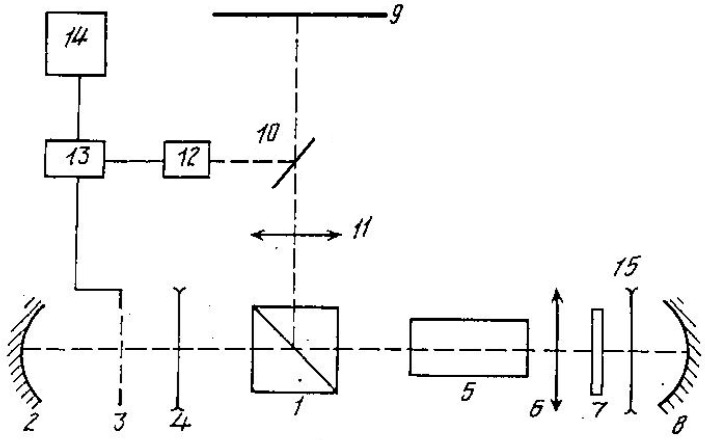
LAOS optical scheme with the polarization separation of strong processing beam [34].

To prevent damage to the mask, schemes were proposed in which a strong processing beam was isolated from the STLM through polarization separation [34] (see Figure 10).

The presented optical scheme incorporates a polarizer (1) positioned in front of the laser medium (5). ASE from the active element (5) passes through the polarizer (1), acquiring linear polarization, and then illuminates the spatial-temporal light modulator (STLM) (3), which generates dynamic information for object processing. After passing through the STLM (3), the ASE is reflected by mirror (2) back into the active medium (5) for double-pass amplification. A quarter-wave plate (λ/4) (7) then rotates the polarization of the light. Upon reflection from the feedback mirror (8) and passing through the quarter-wave plate (7) a second time, the light’s polarization becomes orthogonal to its initial state. This orthogonally polarized beam undergoes a second amplification in the laser medium (5) and is subsequently reflected by the polarizer (1), forming the output processing beam directed onto the object (9).

This configuration enables the extraction of a high-power laser beam from the cavity for object processing, while simultaneously protecting sensitive optical components from damage. As a result, the system is also suitable for macromachining applications.

**Figure 11:**
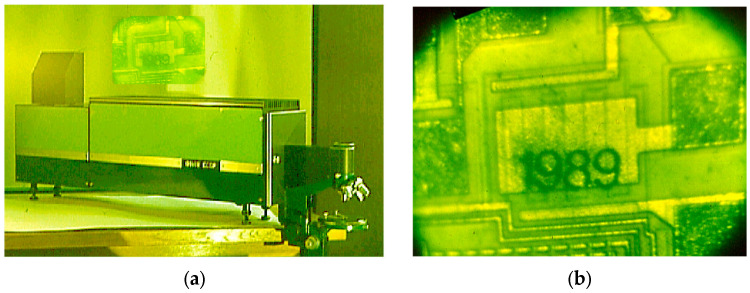
(**a**) Laser projection microscope; (**b**) the result of chip micro-processing.

Several commercial LAOSs based on the optical schemes discussed above have been developed [29,35]. One such system, the LPM, is shown in Figure 11a. This system utilized a CVL amplifier and incorporated a conjugate resonator for the micromachining capabilities, with the results demonstrated in Figure 11b.

The laser projection microscope was most commonly used for laser lithography in microelectronics manufacturing [36,37,38]. Thin layers of materials such as Cr, Al, Cu, Au, iron oxide, and polysilicon could be easily removed from substrates. The process was typically monitored visually using the laser projector screen. In addition, the system was also used for tasks such as trimming electronic components, precision drilling, and more. STLMs served as masks for marking silicon wafers (see Figure 9 and Figure 10). This type of LAOS was also employed for pulsed treatment of semiconductor structures to modify their properties. Common applications included annealing semiconductor layers after ion implantation; processing ‘silicon-on-sapphire’ structures to enhance crystallographic quality; pre-epitaxial treatment of silicon wafers to reduce microdefect concentrations; and laser gettering of semiconductor wafers and various other material modification processes.

Unlike traditional laser processing projection systems, which typically suffer significant light losses at the mask that also can damage them, this system amplifies only the useful beam that carries the mask image. As a result, the masks are safe and mask-related losses are effectively eliminated, and the active medium is utilized far more efficiently. Under typical operating conditions, the active medium in this system functions at a high level of saturation. This enables nearly complete utilization of the stored inversion within the volume occupied by the processing beam. Consequently, the power of the processing beam depends only weakly on the open area of the mask, and the power density on the target is inversely proportional to that area.

The combination of low optical losses, high efficiency, and the ability to focus the processing beam down to micron-scale areas on the target allows the system to achieve average power densities as high as 10^7^ W/cm^2^ and peak power densities up to 10^11^ W/cm^2^. This is accomplished even with laser elements delivering a relatively modest total average power of 5–10 W.

**Figure 12:**
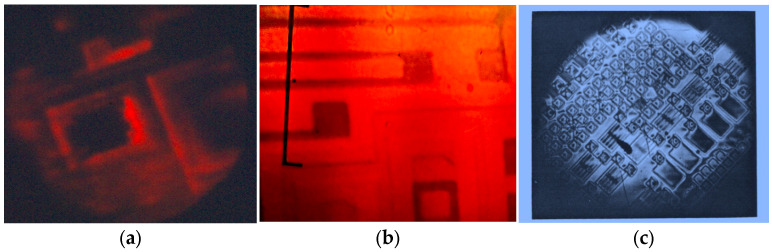
Images of the microchip carried by laser beams of LAOSs with different amplifiers: Ba-amplifier with λ = 1, 5 μm with through pass image of the microchip carried presented (**a**); Au-amplifier with λ = 628 nm (**b**); Sr-amplifier with λ = 430 nm (**c**).

In addition to CVL amplifiers, various other types of laser amplifiers have been utilized in these LAOSs. Examples include the Ba-amplifier (barium) [39,40,41,42] with wavelength λ = 1.5 μm, which transmitted a microchip image carried by a laser beam (Figure 12a). An infrared laser projection microscope (IR LPM) equipped with a large fluorescent screen for visualizing IR images was investigated using a barium vapor brightness amplifier to evaluate its performance characteristics. A notable single-pass gain of approximately 2.7 × 10^3^ was achieved. Among various known classes of phosphors, optimal screens were selected based on their ability to meet the stringent image quality requirements specific to IR LPM applications.

The key performance characteristics of a gold vapor brightness amplifier operating at 627.8 nm were measured within the framework of an LPM setup, as reported in [43,44,45]. A peak effective amplification of approximately 3500 was achieved per single pass through the active element. The system also demonstrated a maximum light output exceeding 0.5 W on the projection screen. Additionally, it was shown that the gold vapor amplifier, when used as a brightness amplifier, is capable of generating a structured laser beam (see Figure 12b).

The potential for image brightness amplification using a strontium vapor active media was highlighted in [46,47]. An image intensification regime was successfully implemented in the violet spectral region, and enhancements in image sharpness and brightness were studied using a multipass, self-conjugate optical system similar to the one depicted in Figure 9. In this setup, the active medium of the Sr amplifier operated at a wavelength of 430 nm (see Figure 12c).

The XeCl excimer laser micromachining projection microscope, operating at a wavelength of 308 nm, enabled high-magnification imaging of a surface while simultaneously offering the capability to micromachine the same surface based on a predefined pattern [48,49]. The laser provided bright illumination through amplified spontaneous emission, along with a focused laser spot delivering sufficient fluence to modify or mark the sample. Key characteristics of image enhancement achieved within the excimer amplifier were presented. This technique demonstrated clear advantages for pattern generation, surface cleaning, and mask correction, as evidenced by results on Al, Cu, and W specimens, achieving spatial resolutions finer than 3 μm.

**Figure 13:**
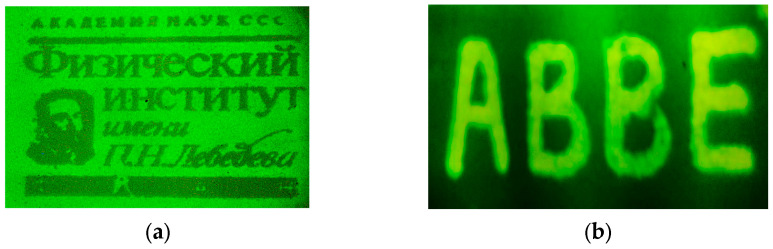
Examples of macromachining with the LAOS device on the base of a CVL amplifier. (**a**) is the logotype of Lebedev Physical Institute, Russia (size 20 × 15 mm); (**b**) Last name of German physicist Ernst Abbe (size 30 × 10 mm).

Additionally, the results of macromachining using a LAOS device based on a CVL amplifier and a scheme similar to the one shown in Figure 10 [34,50,51] are illustrated in Figure 13. Large-scale images, several centimeters in size, were created on glass substrates coated with various dielectric and metal films.

**Figure 14:**
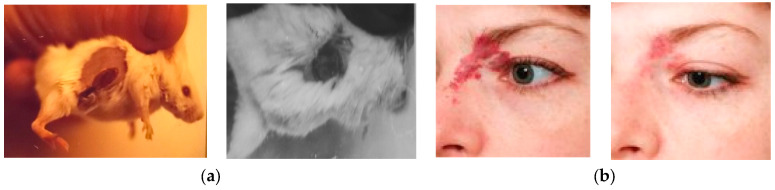
LAOS applications for the biology and medicine: (**a**) mouse oncological photodynamic therapy with the gold vapor laser system, with and without beam shaping; (**b**) before and after port-wine stain treatment with the copper vapor LAOS.

LAOSs have also been applied in biology and medicine, in experimental research and clinical practice [52,53,54,55,56]. These systems enhance treatment effectiveness and selectivity, particularly in cases where pathological tissues have complex shapes surrounding healthy ones, as often occurs in oncology and cosmetology. Figure 14 illustrates examples of such treatments. Figure 14a illustrates photodynamic therapy [52] for oncology in a mouse model using a gold vapor laser system, comparing treatments with and without beam shaping [53,54].

Figure 14b presents before-and-after images of port-wine stain treatment performed with a copper vapor LAOS.

The most recent investigations and applications of LAOSs were presented in [57,58,59,60,61,62,63,64,65,66,67,68,69,70,71,72]. LAOSs were used for non-destructive testing [59,60,61,62] of materials and processes shielded by intense background lighting [57,58,59,60,61,62]. Particularly, these studies discussed bistatic laser active optical systems, which incorporate two active elements: an illumination source and an optical signal converter (brightness amplifier) with synchronized pumping sources. Both the illumination source and the brightness amplifier are based on active media in copper bromide vapors [63,64,65]. These devices can capture the imaging of processes occurring at long distances, even when obscured by background light with a brightness temperature of up to 45,000 K and a temporal resolution of up to 10^−5^ s. This capability enables the study of advanced manufacturing processes, such as the formation of new nanomaterials and surface modifications. Examples include reactions on the surface of carbon electrodes at approximately 4000 K, laser metal welding, self-propagating high-temperature synthesis, and nano-powder production.

Extensive efforts were dedicated to a deeper investigation of image brightness formation and amplification. Based on these findings, an improved version of LAOSs with enhanced parameters, along with novel micro processing techniques using specially designed laser beams, was proposed [67,68,69,70,71]. Additionally, a semi-empirical mathematical model of the brightness amplifier, based on self-terminating transitions in laser active media, was applied to the copper bromide vapor brightness amplifier [72]. This model enabled the distinction between amplified spontaneous emission and the amplified input optical signal, allowing for the study of gain and the theoretical maximum contrast of the brightness amplifier as a function of the temporal and energy characteristics of the input optical signal.

## 3. LAOSs with Solid-State Disk-Shaped Crystal Amplifiers

**Figure 15:**
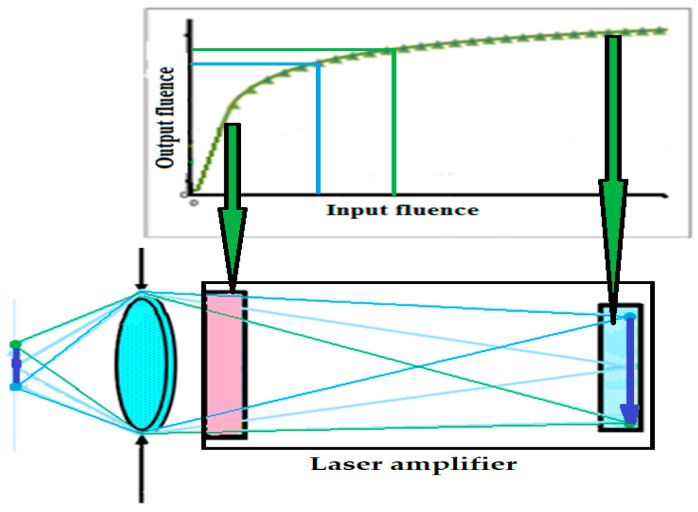
In a LAOS with rod-like amplifiers, the beams carrying different points of image in active media are separated at the output (compare the blue and pink areas).

The practical LAOSs discussed above were primarily limited to the use of metal vapor amplifiers, which constrained the output average power to a few watts and the pulse energy to a few millijoules. Additionally, the number of pixels forming the image was restricted to below 1 megapixel due to the low aspect ratio of tube- or rod-shaped amplifiers. Furthermore, in active media with this geometry, beams originating from different points of the object (see green, light blue, and dark blue lines) tend to diverge at the output of the active element (blue area in Figure 15), potentially experiencing different gain levels. If a high-intensity input signal undergoes low gain while a low-intensity input experiences high gain, the output signals may become equal. In contrast, in the pink area, beams from different object points are well mixed, resulting in uniform gain and preservation of the original input signal ratio. For effective operation, the active medium must reach saturation, which results in a loss of image contrast with this beams separation. This effect is described by the equation *g = g*_0_*/(*1 *+ I/I_s_),* where *g* and *g_0_* are the gain and small signal gain, respectively, and *I* and *I_s_* are the input intensity and saturated intensity, respectively, demonstrated by the curve in Figure 15. Here, the difference in output intensities becomes smaller than in the input ones produced by different object points (compare the blue and green lines on the graph).

In a single-pass configuration similar to that of a copper vapor laser with a rod-like active element geometry, the region where beams from various points of the object are well mixed (pink on the figure) is situated near the entrance of the amplifier. Saturated gain in this region does not degrade image quality. However, this configuration inherently supports only linear gain in this area.

These limitations were overcome by replacing the rod-shaped amplifiers with disk or thin-disk (TD) laser media. The required reduction in the length of the active medium for a single-pass transmission was compensated by multipass gain propagation and/or multistage amplification [73]. This technology allows for confining the area where spatial harmonics of the image are effectively mixed in an amplifier. This area can be transmitted from pass to pass and from stage to stage by relaying optics. Moreover, in such schemes, gas active media with uniformity of population inversion are no longer required because the output intensity of each point of the image is determined by the light passing through the full aperture of the amplifier. As a result, the output image contrast replicates the input one. Moreover, this technique offers significant potential to broaden the range of achievable power levels, pulse energies, durations, and operational wavelengths, as it allows for the use of various types of laser active media in LAOSs, including solid-state materials.

It has been demonstrated that TD-amplifiers can increase average power to hundreds of kW [74] using Nd:YAG and Yb:YAG laser crystals [75,76,77,78,79,80,81,82]. The thin-disk laser technology, with diode-laser pumping (efficiency up to 60%), is well-suited, offering advantages such as high repetition rates (kHz), high average power (Nd:YAG, Yb:YAG–up to 100s of kW in CW mode), and hundreds of millijoules of energy with nanosecond pulse durations.

**Figure 16:**
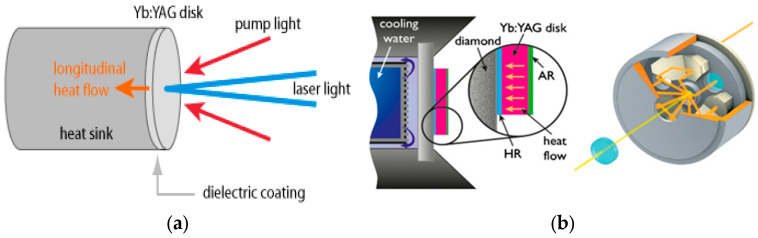
Optical scheme (**a**) and construction (**b**) of Yb: YAG TD amplifier [64].

Figure 16 illustrates the optical scheme (a) and construction (b) of the Yb:YAG thin-disk (TD) amplifier, which enables highly efficient heat extraction.

**Figure 17:**
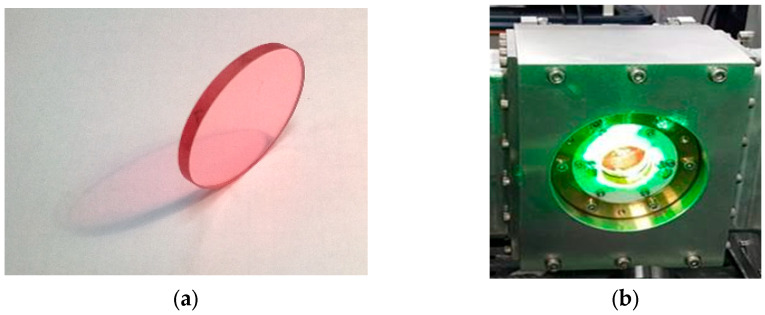
Ti:Sapphire crystal amplifier: (**a**) Ti:Sa crystal of 35 mm Ø × 3 mm; (**b**) room temperature water-cooled crystal in the mount pumped by Nd:YAG laser with λ = 532 nm [69].

Another promising medium tested for LAOS applications is the TD titanium sapphire (Ti:Sa) amplifier (see Figure 17) [82,83,84,85,86,87,88,89,90,91,92]. In these experiments, a crystal measuring 35 mm in diameter and 3 mm in thickness was pumped with 5 J pulses at λ = 532. After three amplification passes, the seed pulses reached an output of 2.6 J, with a compressed pulse duration of less than 30 fs [86]. The development of Ti:Sa thin-disk (TD) amplifiers became feasible through the integration of extraction during pumping (EDP) schemes [83,84,85] with thin-disk technology. This combination effectively addressed challenges related to crystal thermal management and transverse amplified spontaneous emission (ASE), enabling the realization of high average power laser systems based on TD Ti:Sa amplifiers.

This technology was further advanced in [87], where an output pulse energy of 0.3 J at a 100 Hz repetition rate was demonstrated. In general, laser systems utilizing Ti:Sa crystals can significantly reduce the number of amplification passes while shortening pulse durations to tens of femtoseconds, increasing peak power to the petawatt (PW) level, and achieving pulse energies in the hundreds of joules. The application of thin-disk (TD) crystal geometry also enables higher repetition rates, reaching the kilohertz (kHz) range [91,92], and achieving average output power in the kilowatt (kW) range.

**Figure 18:**
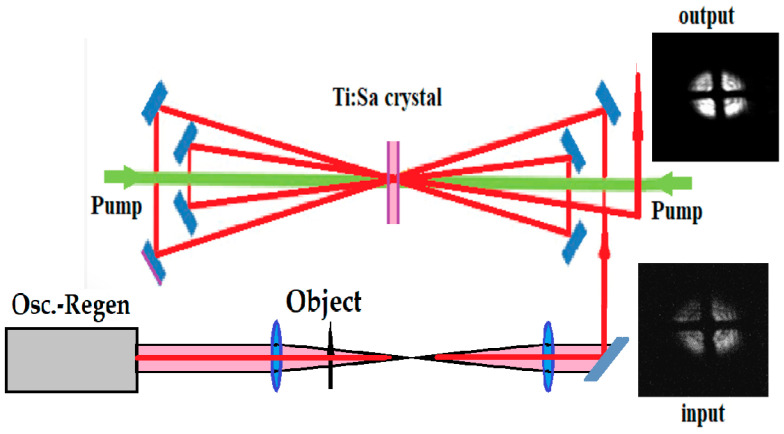
Schematic diagram of the LAOS with Ti:Sapphire crystal amplifier (Modified from [73]).

The Ti:Sa laser system was tested as a LAOS in [73]. The optical scheme is presented in Figure 18, where the oscillator-regenerative amplifier system generated ns pulses with a repetition rate of 10 Hz was used as a pulsed light source for object illumination. After passing through the regenerative amplifier, the beam traveled through a 1:1 telescope, with the object positioned near the focal zone. Following the telescope, the image-carrying beam was amplified in a four-pass Ti:Sa amplifier, which had a pump area diameter and thickness of 1 cm each.

After attenuation, the amplified image was transmitted to a CCD camera and powermeter. A mesh with a wire diameter of 14 μm was used as the object. The laser beam, carrying the image with an initial pulse energy of 0.2 mJ and a duration of 7 ns, was amplified by a factor of 80, reaching 16 mJ. The images before and after amplification are shown in Figure 18. This experiment demonstrated the capability of multipass solid-state disk-shaped laser media to amplify image brightness with minimal distortion and a gain close to two orders of magnitude. The optical setup used was the front-end section of a system based on chirped pulse amplification (CPA) technology, which has the potential to significantly reduce pulse durations to just a few femtoseconds. CPA technology, introduced in 1985, was developed to shorten pulse durations and boost peak power [93,94]. Recent advancements in CPA laser systems have enabled the achievement of remarkable peak powers, reaching up to 10 PW (10^15^ W) [95,96].

To further increase the output energy, multiple similar amplifiers with higher pump energy can be arranged in a series, with image relaying and magnification implemented between stages. The pulse repetition rate or average power can also be enhanced by using thinner Ti:Sa crystals (see, for example, [86,87,88]).

## 4. Conclusions

This review presents a retrospective analysis of the development of laser active optical systems (LAOSs). These laser systems enabled the generation of laser beams with precisely controlled energy distributions across the aperture, making them suitable for material processing. The first successful implementation was demonstrated in the 1970s using metal vapor lasers as gain media. In these systems, material processing was achieved either by a beam with a predefined energy profile or by projecting the image of the object itself. Various laser media have been utilized for LAOSs, including metal vapors, excimer XeCl lasers, and solid-state media.

Several modifications were introduced during the development of these systems, including the integration of Space-Time Light Modulators (STLMs) and CCD cameras into the optical scheme, the use of multipass amplifiers, and the adoption of disk or thin-disk (TD) solid-state laser amplifiers. These advancements have significantly expanded the range of power, energy, pulse durations, and operational wavelengths.

Modern TD laser amplifiers and STLM DMD technology enable the development of various LAOS devices for both Additive and Subtractive Manufacturing (AT, ST) in macro- and micro-nano processing. Additionally, LAOSs can be effectively applied in medicine to enhance the selectivity of laser treatments. Advances in disk and thin-disk laser media, such as Nd:YAG, Yb:YAG, and Ti:Sa pumped by diode lasers, along with significant progress in STLM and CCD technology, have facilitated the development of LAOSs capable of meeting the stringent demands of the AT and ST industries while expanding their potential applications.

In general, LAOSs have the potential to drive significant advancements in laser materials processing. They aim to develop a new class of intelligent laser-imaging systems for technological innovation by enabling the processing of various surface and bulk materials under remarkable parameters such as TW/cm^2^ intensity, kW-level average power, pulse durations ranging from 10 fs to continuous wave, Tpx/s processing speeds, and nanometer-scale resolution. Beyond subtractive manufacturing, modification, and repair of components, these systems could also enhance additive technologies, significantly boosting production speed and flexibility. Therefore, one of the goals of this review is to draw the attention of the scientific and engineering community to these promising laser systems for material processing, encouraging further research and development.

## Figures and Tables

**Table 1:** The main parameters of the manufactured lasers active elements.

Devices	Total Average Output power, W	Beam Diameter, mm	Repet. Rate, kHz	Consumed Power kW	
**1. Active elements**				Rectifier	Wall plug
Kulon-Sil	1–1.5	7	8–30	0.5	0.6
GL-204M	4–6	12	8–30	1.0	1.2
GL-202M	5–8	12	8–30	1.8	2.2
Kulon-S	8–12	12	8–30	1.4	1.7
UL-102M *	8–12	20	5–20	1.8	2.2
GL-201M	25–35	20	5–20	3.0	3.6
GL-201D	35–45	20	5–20	4.0	4.8
Crystal-32D	45–55	32	5–15	5.0	6.0
**2. Laser**					
Columbia-L	3–5	12	8–18	1.3	1.5
LGI-101M	5–8	12	8–12	2.5	2.8
LGI-202M	25–30	20	8–12	4.0	4.5

* The main rise of UL-102 is image brightness amplification in active projection systems.

**Table 2:** Parameters of metal vapor brightness amplifiers.

Active Media	Wavelength, nm	Rep. Rate kHz	N, *10^5^	Small Signal Gain	P_am_/P_l a_ **	Measured eff. Amplification ***
**Cu**	510.5	10	12	0.2	0.44	1.6 × 10^4^
**CuCl**	510.5	10	32		0.56	
**CuBr**	510.5	14	32		0.59	6.5 × 10^3^
**Au**	627.8	10	5.4	0.11	0.34	10^4^
**Mn**	green	7	5.7	0.11	0.18	10^3^
**Mn**	IR	7	1.1	0.13	0.2	3.5 × 10^3^
**Pb**	722.9	12	3.1	0.12	0.52	3 × 10^3^
**Ba**	1500	8	2.1	0.17	0.82	2.7 × 10^3^

In this table: * *N = (D*^2^*/λL)*^2^ represents the number of pixels over the field of view with diffraction-limited optics (where *L* and *D* are the tube length and diameter, respectively); ** P_am_/P_la_ is the ratio of the amplifier’s average output power to the average output power of a laser using the same amplifier with a flat-flat resonator and an uncovered quartz plate as the output mirror; and *** effective amplification refers to the ratio of average power at the input and output of the amplifier.

## Data Availability

There is no research data for a sharing.

## Glossary of Terms

LAOSs	Laser Active Optical Systems
OSs	Optical Systems
STLMs	Space-Time Light Modulators
DLP	Digital Light Processor
DMDs	Digital Micromirror Devices
CCD	Charge-Coupled Device
ST	Subtractive Technology
AT	Additive Technology
CW	Continuous Wave regime
TD	Thin-Disk crystal
MVL	Metal Vapor Laser
CVL	Copper Vapor Laser
LPM	Laser Projection Microscope
ASE	Amplified Spontaneous Emission
Ti:Sa	Sapphire doped by Titanium
Nd:YAG, Yb:YAG	Yttrium Aluminum Garnet doped by Neodymium or Ytterbium
EDP	Extraction During Pumping method of amplification
CPA	Chirped Pulse Amplification method
kW, TW, PW,	Kilowatt, Terawatt, Petawatt,
TPx	Tera Pixel
